# Integration degree of China’s the new development pattern of dual circulation and industrial green development

**DOI:** 10.1371/journal.pone.0288160

**Published:** 2023-07-07

**Authors:** Jianlin He

**Affiliations:** School of Economics,YunnanUniversityofFinanceandEconomics, Kunming, Yunnan, China; Iqra University, PAKISTAN

## Abstract

The long-standing development pattern dominated by international circulation makes China face the risk of "low-end lock-in" and "being decoupling". In addition, the current global climate change and environmental crisis are becoming increasingly severe, coupled with the COVID-19 impact. Chinese enterprises must actively build a green development system for domestic industries in a mutually reinforcing manner in the context of the domestic general circulation, in order to adapt to the new dual circulation environment as soon as possible. This paper analyzes the specific coupling and coordination relationship between the two systems based on the relevant data of China’s three major industries from 2008–2014 using Index DEA, entropy value method, gray correlation analysis and coupling coordination model. The results of the study show that: the two systems of dual circulation pattern and industrial green development have a strong correlation and basically present a coupling relationship, but within the industry, there is a problem of the collapse of the tertiary industry. In terms of the type of coupling, the domestic and international circulation in general gradually present the green development leading state, except for the primary industrial segment of the international circulation. On the whole, the coupling quality of the two systems needs to be further improved. Based on this, this paper puts forward the following suggestions: (1) coordinate the internal and external development of the industry; (2) take innovation as the driving force to promote the green transformation of industries; (3) take green sharing as the goal to strengthen the policy orientation of green development; (4) take the opportunity of mutual promotion of dual circulation to stabilize the steady state of green development coupling.

## Introduction

As a result of the global financial crisis in 2008, the international market shrank significantly and external demand weakened, which in turn weakened the momentum of the international general circulation [1]. Coupled with the rise of trade protectionism in Western developed countries, escalating economic and trade friction between China and the United States, theemergence of anti-globalization thinking, and the rapid spread of the new crown pneumonia epidemic worldwide in recent years, these external environments have posed increasinglycomplex challenges to China’s development situation. Various risks and uncertainties have increased accordingly, and the security and stability of global industrial and supply chains are facing many challenges [2]. To cope with the intricate external environment and changes in the situation, President Xi Jinping proposed to promote a new development pattern by taking the major domestic circulation as the mainstay and promoting each other with the domestic and international circulation [3].

In the face of the new development pattern of dual circulation, China’s internal and external supply chain and value chain structure will usher in new changes. China’s long-term international-circulation-oriented development pattern has made its disadvantage in the global competition and division of labor system more and more obvious, which is mainly reflected in two aspects: first, the "low-end lock-in" effect [4]. Due to the high energy consumption, high pollution and over-reliance on external technology, Chinese enterprises have long faced green technology barriers and environmental barriers from developed countries. Second, the risk of "being decoupled" [5]. The crude single production chain leads to the lack of irreplaceability of Chinese enterprises in GSCs and value chains, and the linear development pattern of inputs and outputs, which makes China easily "decoupled" from GSCs and value chains. In addition, the current global climate change and environmental crisis are becoming common concerns around the world, coupled with the impact of the COVID-19 pandemic, GSCs and value chains have shifted to place more emphasis on green and safety, and countries have learned from the pandemic to improve the layout of their green supply chains and raise the green barriers for other countries’ products to enter their countries. These will increase the risk of Chinese companies being "decoupled" in the coming period.

In the face of these problems, Chinese enterprises must accelerate the pace of green transformation in the context of the large domestic circulation, adhere to the inward force, actively build domestic green supply chains and green value chains in a mutually reinforcing manner, fundamentally break through the international-circulation-dominated supply chains and value chains, and adapt to the new dual circulation environment as soon as possible.

## Literature review

### The new development pattern of dual circulation

A review of the existing literature reveals that academic research on the new development pattern of dual circulation began in May 2020. Before the Fifth Plenary Session of the 19th CPC Central Committee, academics focused on the internal logic, theoretical mechanisms and policy interpretation of the dual circulation [6–9]. After the Session, the academic community focused more on the analysis of industrial digital transformation, industrial development paths and global value chain reconfiguration of manufacturing industries in the context of dual circulation [10–12].

In terms of its scientific connotation, academics generally agree that domestic and international circulation are dialectically unified [7, 9], with the former in the main position. In other words, China should take the expansion of domestic demand as the starting point and objective of economic development, give full play to its market advantages and domestic demand potential, and participate in international circulation at a higher level [7]. At the same time, through international circulation, it can fully utilize two markets and two resources to improve efficiency. When international circulation is risky, domestic circulation can ensure the stability and security of China’s economic development [9].

As for the practical reasons for the construction of the new development pattern, in addition to the changes in the international economic and trade situation, there are also problems that have accumulated internally in the course of China’s long-term development. First, the "export-oriented" economic model is not conducive to the development of the current Chinese economy, which is mainly reflected in the diminishing demographic dividend [13], insufficient domestic demand [14], and the curtailment of core technology development [15], etc. Second, the new development pattern is proposed as an urgent need to deepen the structural reform of the economy and promote high-quality economic development. At present, China’s economic and social development faces many problems, such as an unreasonable ratio of industrial composition [16], insufficient independent innovation capacity [17] and a widening income gap among residents [7]. Building a new development pattern of dual circulation is advantageous to deepening China’s domestic reform [15], providing theoretical guidance and new ideas for solving the difficulties and dilemmas encountered in China, and pointing out a new direction for socialist economic construction with Chinese characteristics [9].

With regard to the research on the practice path of dual circulation, scholars have mainly studied how to promote the formation of the new development pattern from each link of reproduction activities, i.e., investment, production, distribution, circulation, and consumption [7, 9, 13, 18, 19]. In promoting domestic and international dual circulation, scholars mainly think from the perspectives of opening up to the outside world, guarantee mechanisms and governance systems [7, 16, 20].

The relevant research has achieved certain results through the multi-directional sorting and interpretation of scholars in related fields. However, due to the high convergence of research in academic circles, most of them are generalized argumentation and interpretation of policy documents, lacking in problem awareness and academic rationality, and there is a relative dearth of research in quantitative aspects such as measurement and empirical data, which requires a further deepening of relevant research.

### Green development

The new development pattern reflects a new market environment, which requires a smooth domestic and international dual circulation, so the green transformation of the industrial structure becomes an important way to promote economic growth. The upgrade of industrial structure can reduce highly polluting industries and promote green economic development. Green development is particularly important to China today, and in accordance with the spirit of the 19th National Congress of the CPC, building a new development pattern and implementing a dual carbon policy are measures to promote green development. Green development can reduce pollution and achieve green growth, and is also a requirement for industrial structure upgrading.

Internationally, green development is usually expressed by indicators such as "green economy" and "green growth". The concept of "green economy" was first introduced by Pearce. Ji defines the green economy as an economic structure, growth mode and social form with efficiency, harmony and sustainability as the development goals and ecological agriculture, recycling industry and sustainable service industry as the basic contents [21]. The Green New Deal and the green development of industry have received a lot of attention in recent years. UNEP and Rio+20 earth summit both highlight the importance of implementing a Green New Deal [22]. In addition, the G20 Summit was held on November 15, 2015, which proposed "adhering to green and low-carbon development". Subsequently, the APEC meeting advocated inter-regional cooperation and innovation to strengthen the green economy.

"Carbon peaking" and "carbon neutrality" are the focus of China’s green development efforts. To promote green development, we need to green our production and lifestyle. In terms of how to effectively reduce carbon emissions to achieve green development, a study by Wang et al. showed that rapid and large-scale deployment of clean energy plays a significant role in reducing carbon emissions in China [23]. Sun et al. found that recycling of municipal solid waste and energy efficiency contribute to reducing carbon emissions and other greenhouse gases, thereby improving environmental performance and safeguarding natural resources [24]. In addition, Bashir et al. stated that although increasing population size and economic growth can significantly lead to environmental degradation, natural resource rents, electricity transition, and human development contribute to green and sustainable development [25]. At the governmental level, Anu et al. pointed out that the government should develop appropriate policies to finance renewable and clean energy projects and form a policy-oriented and financial service system that is conducive to promoting the green transition. At the same time, the paper suggested at the enterprise level that enterprises should use green technologies and change their production methods and management models to achieve green production [26]. At the investment level, Raza et al. point out that non-renewable energy PPP investments increase carbon emissions, which leads to environmental degradation. Therefore, there is a need to shift PPP investments to more efficient renewable energy sources [27]. At the resident level, Liao et al. argued that the public needs to develop an awareness of green consumption, conservation and environmental protection. The government, enterprises, and residents make concerted efforts, and only in this way can we promote the green development of the whole society [28].

Academics have conducted in-depth research on the connotation and mode of green development and other aspects, and have achieved greater results, but there are problems in the research on the energy mechanism of green development and other aspects in the context of economic structural transformation. The research on the impact of industrial structure adjustment and economic growth mode transformation on carbon emission needs to be deepened.

### Dual circulation and industrial green development

From the existing studies, scholars have noted the coordinated interaction between the new dual circulation development pattern and industrial green development. Zeng and Huang emphasized that a green financial system based on domestic circulation is a key measure to alleviate many environmental problems caused by China’s development in recent decades, thus achieving the goal of carbon neutrality and a low-carbon sustainable development path [29]. Chen et al. analyzed that, under the dual circulation development pattern, enterprises strengthen their efforts to create shared value, actively cooperate with domestic suppliers to implement green procurement and promote green consumption on the premise of achieving common benefits can help to break the blockage of the "inner circle" of the green economy in all aspects and facilitate the participation of enterprises in global environmental governance [30].

Some scholars have also studied the path of industrial green development under dual circulation. A study by Liu and Shen pointed out that the construction of the inner-loop green supply chain should focus on greening supply-side reform, and the key to the outer-loop green supply chain is to improve the quality of international green factor supply and develop the demand for green products in the international market. Meanwhile, the construction of green value chain should coordinate the R&D and transfer of green technology at home and abroad [12]. Jie and Ning comprehensively analyzed the greening trend of small and medium-sized manufacturing enterprises in the dual circulation environment, and concluded that only by strengthening independent green innovation capability and increasing green investment can enterprises better face the dual circulation and continuously build their own green advantages [31].

From a certain perspective, the dual circulation pattern and industrial green development are both independent systems, but they also embody mutually integrated and complementary dynamics. Existing studies focus on green development based on the industrial level to support the construction of the dual circulation pattern, but the embodiment and portrayal at the industrial level are obviously insufficient, and the discussion dimension is mostly a single industrial perspective, lacking multi-industry analysis under the global view. In addition, most scholars have noticed the path of industrial green development in the dual circulation pattern, but the mutual coupling mechanism between the two systems has not been proved and analyzed by quantitative methods.

Therefore, the marginal contribution of this paper is to reveal the intrinsic linkage and coupling mechanism between the two systems from both domestic and international perspectives by selecting more comprehensive evaluation indicators, and making policy recommendations on this basis.

## Analysis of the coupling mechanism

**Fig 1:**
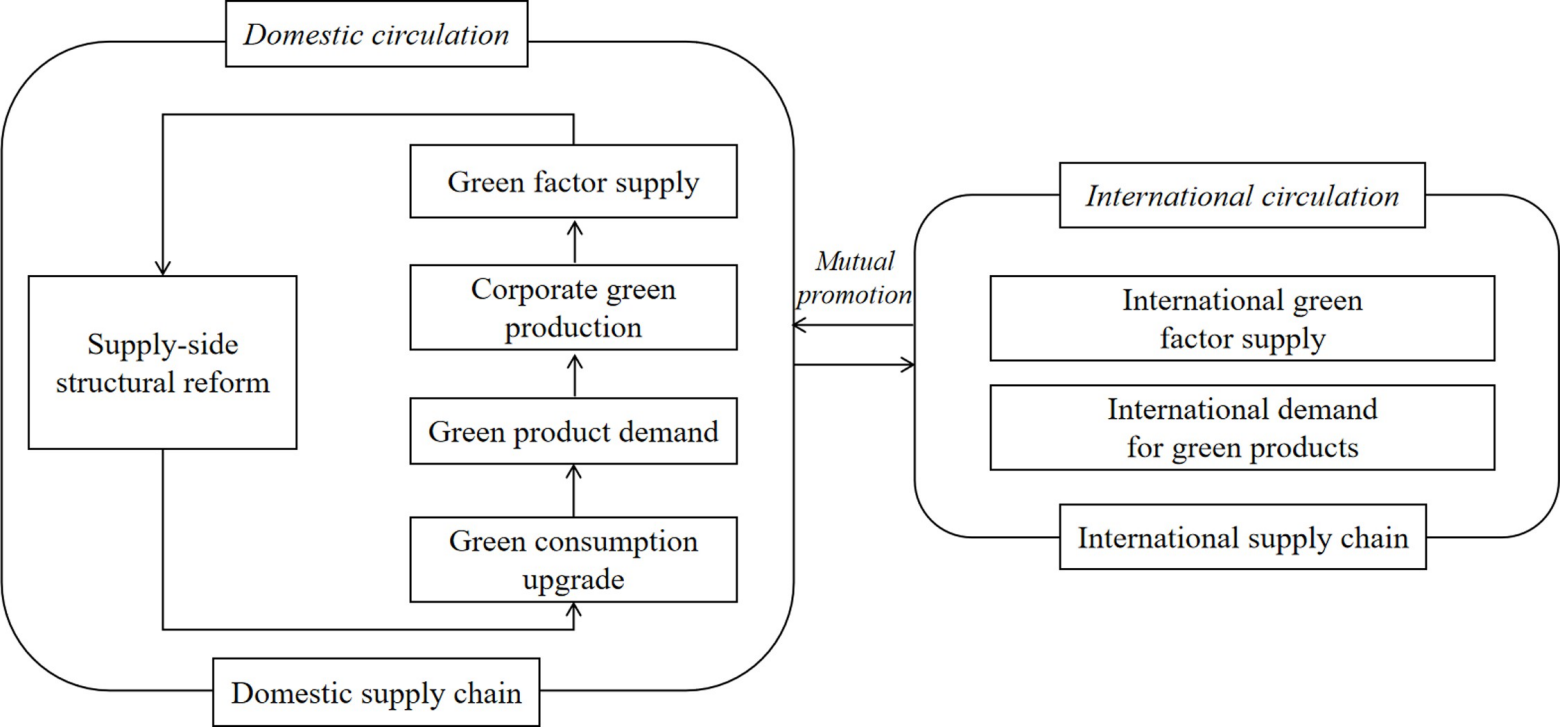
Dual circulation pattern and industrial green development coupling system.

In terms of the relationship between the two systems, as shown in Fig 1, the dual circulation provide opportunities for industrial green development. At the same time, the green development of the industry can also further promote the sustainable operation of the domestic and international circulation.

In the process of the domestic grand circulation, the overall aim is to build a green and sustainable production and circulation system, thereby opening up the blockage of domestic circulation. The key to domestic green circulation lies in the structural reform on the supply side and the reform on the demand side. The supply-side reform focusing on improving the quality and efficiency of green supply as well as the greening level of industry leads and creates green consumption upgrade, which in turn promotes demand-side reform. The increase in consumer demand for green products will force enterprises to carry out green production, and in the process will prompt the green supply of factor resources. When it is transferred to the supply-side structural reform, it will prompt a new round of greening. The whole process builds up an interlocking domestic green circulation system, which goes through all aspects of production, distribution and consumption.

In terms of international circulation, firstly, the industrial green development system built up with the help of domestic large circulation can well assist the efficiency of international circulation and improve the quality of international green factor supply. Secondly, relying on the international circular supply chain, China can carry out green product export and use it to develop the international green product demand market and layout a China-led international green supply chain system. In addition, integrating the concept of environmental protection or ecology into the whole process of China’s foreign trade can promote the ecological transformation of China’s international trade, build a green international value chain, and form a unique new advantage in international competition.

## Methodology

### Connotation of relevant quantitative measures

#### Indicator constructions

Industrial green development presents internal system differentiation in terms of structure and dimensional factors, and the connotation presents different forms due to the different natures of industries. The research and analysis should be evaluated objectively based on a multi-dimensional index system to obtain a more realistic status.

In this paper, combined with the definition of the connotation of industrial green development and the analysis of the development status of each industry in China, an index evaluation system suitable for the actual situation in China is constructed. Specifically, the evaluation indexes of green development under each industry are subdivided from the perspective of three major industries [32, 33], as shown in Table 1.

**Table 1:** Industry green development evaluation index system.

First-Level Index	Second-Level Index	Unit	Nature of indicators	Weights
Primary Industry	Land yield rate	%	+	0.1507
	The proportion of water-saving irrigation area to the effective irrigation area	%	+	0.1680
	Soil and water conservation and ecological projects completed investment	Billion	+	0.1589
	Fertilizer use per unit area	Tons/thousand hectares	-	0.2891
	Pesticide use per unit of arable land area	Tons/thousand hectares	-	0.2332
Secondary Industry	Secondary industry Labor productivity	10,000 Yuan/person	+	0.1016
	Sulfur dioxide emissions per unit area	million tons	-	0.0937
	Energy consumption per unit of industrial added value	Ton of standard coal / million yuan	-	0.1256
	Water consumption per unit of industrial added value	Cubic meter/yuan	-	0.1133
	Total wastewater per unit of GDP	Ton/yuan	-	0.1042
	Solid emissions per unit of gross regional product (dumped and discarded)	million tons	-	0.1257
	Comprehensive utilization of industrial solid waste	million tons	+	0.0659
	Industrial pollution control completed investment	Billion	+	0.2010
	Green technology innovation input	Billion	+	0.0658
Tertiary Industry	Tertiary industry labor productivity	Yuan/person	+	0.3136
	The proportion of tertiary industry output value to regional GDP	%	+	0.3101
	The proportion of employed persons in the tertiary sector	%	+	0.3763

#### The connotation of the dual circulation

**Fig 2:**
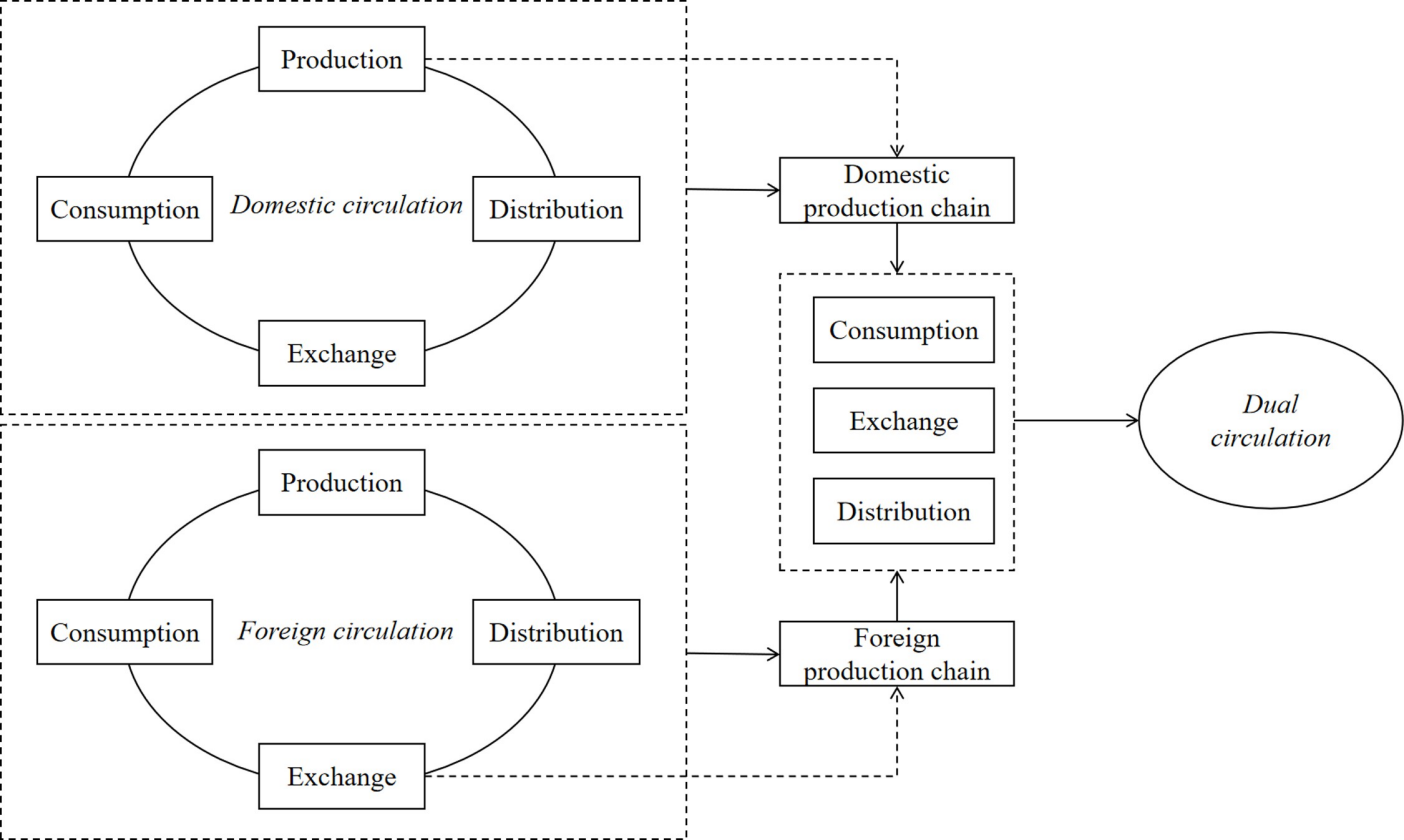
Dual circulation reproduction mechanism.

The dual circulation is characterized as a new Marxist theory of social reproduction. Based on this, the paper builds a complete framework for the operation of dual circulation, as shown in Fig 2. This pattern embodies the four cycles of production, distribution, exchange and consumption involved in the form of social reproduction from the perspective of domestic circulation and international circulation respectively. In terms of reproduction mechanism, the nature and changes in the production domain of final products, i.e., changes in the distribution, exchange, and consumption fields of products, can be characterized as the extent of domestic and international circulation.

Domestic circulation degree refers to the number of times that the distribution, exchange and consumption links in the product production process stay in China on the premise of meeting the domestic market and improving the domestic added value. International circulation refers to the number of times that various links in the product production process stay abroad on the premise of satisfying the foreign market and increasing the domestic added value [34].

### Research methods

#### Index DEA

Given that industrial green development evaluation index is a complex endogenous index, this paper draws on Cherchye et al.’s method to apply Index DEA model to the standardized indicator variables [35]. The formula of the model is

$$MaxU_{i}=\sum_{j=1}^{r}\mu_{ij}\cdot y_{ij}$$

$$\sum_{j=1}^{r}\mu_{ij}\cdot y_{ij}\leq 1,i=1,2,3$$

μ≥0,j=1,…,r (1)

Where *U*_*i*_ is the green development degree of the industry, *i* denotes an industry of the three industries, *r* denotes the total number of indicators, and *j* denotes the *j*th indicator in the evaluation index of the green development of the industry. *μ*_*ij*_ is the weight value of the*j*th indicator of the *i*th industry, the *y*_*ij*_ is the value of the *j*th indicator of the *i*th industry.

In order to avoid the subjectivity of assignment, this paper uses the entropy value method to deal with the difference values of the initial indicators and further optimize the final indicator weight values [36]. The specific steps of its formula are as follows.

First, given that the metric systems studied in this paper are all positive values, the raw data are processed using a positive normalization method.


$$y_{ij}^{*}=\frac{x_{ij}-min\left(x_{j}\right)}{max\left(x_{j}\right)-min\left(x_{j}\right)}$$ (2)

Second, in order to avoid the interference of the standardized 0 indicators to the study values, let $y_{ij}=y_{ij}^{*}+10^{-4}$, a more reasonable formula for calculating the characteristic weight of the indicator is obtained.


$$f_{ij}=\frac{y_{ij}}{\sum_{i=1}^{r}y_{ij}}$$ (3)

Third, then based on the information entropy *e*_*j*_ and redundancy degree *d*_*j*_, the final weights of each index *w*_*j*_ are obtained.


$$e_{j}=-\frac{1}{\ln\;(m)}\sum_{i=1}^{m}f_{ij}\ln\left(f_{ij}\right)$$

$$d_{j}=1-e_{j}$$

$$w_{j}=\frac{d_{j}}{\sum {}_{j=1}^{m}d_{j}}$$ (4)

In addition, since in the data obtained from this statistic, the magnitude of the domestic and foreign circulation is not between [0, 1], the data are coupled after normalized counting by the entropy value method; and, since some data result in 0 after normalization, they are approximated to the minimum value of this group of data.

#### Circulation degree measurement model

Referring to the practice of Wang et al. [37], on the basis of combining the production and trade relations in the world input-output table, China’s domestic added value is decomposed. The specific formula is as follows:

$$\hat{V}B\hat{Y}=\hat{V}L{\hat{Y}}^{D}+\hat{V}L{\hat{Y}}^{F}+\hat{V}LA^{F}B\hat{Y}=\hat{V}L{\hat{Y}}^{D}+\hat{V}L{\hat{Y}}^{F}+\hat{V}LA^{F}L{\hat{Y}}^{D}+\hat{V}LA^{F}\left(B\hat{Y}-L{\hat{Y}}^{D}\right)$$ (5)

In Eq (5), *B* = (*I* ‒ *A*)^-1^can be obtained based on the classical Leontief equation, where *A* denotes the input coefficient matrix of 35 industries in 64 economies. Based on the local Leontief inverse matrix, *L* = (*I* ‒ *A*^*D*^)^-1^ can be derived, where *A*^*D*^ denotes the domestic input coefficient matrix, and *A*^*F*^ denotes the import input coefficient matrix [38]. *Y*^*D*^ denotes the final use matrix for meeting domestic demand, and *Y*^*F*^ denotes the final use matrix for meeting foreign demand. *V* denotes the value added coefficient matrix, and $\hat{V}$ is the*V* the diagonal matrix.

The domestic value added involving global trade activities is related to exports of intermediate goods, which mainly include the exports of intermediate goods directly absorbed by the importing countries, the exports of intermediate goods re-exported to third countries, and the exports of intermediate products sold back to the country. Therefore, the total length of domestic value added production is divided into domestic production length and foreign production length, where the former represents the degree of domestic circulation and the latter represents the degree of international circulation. Based on this, the domestic value added can be expressed as:

$$VY_{GVC}=\hat{V}A^{F}\hat{Y}+\hat{V}A^{D}A^{F}\hat{Y}+\hat{V}A^{F}A\hat{Y}+\hat{V}A^{D}A^{D}A^{F}\hat{Y}+\hat{V}A^{D}A^{F}A\hat{Y}+\hat{V}A^{F}AA\hat{V}=\hat{V}LA^{F}B\hat{Y}$$ (6)

Setting the length of domestic production as the weight, the domestic output induced by domestic value added can be expressed as:

$$Xd_{GVC}=\hat{V}A^{F}\hat{Y}+2\hat{V}A^{D}A^{F}\hat{Y}+\hat{V}A^{F}A\hat{Y}+3\hat{V}A^{D}A^{D}A^{F}\hat{Y}+2\hat{V}A^{D}A^{F}A\hat{Y}+\hat{V}A^{F}AA\hat{V}+\cdots =\hat{V}LLA^{F}B\hat{Y}$$ (7)

The foreign output induced by domestic value added can be expressed as:

$$Xi_{GVC}=\hat{V}A^{F}\hat{Y}+\hat{V}A^{D}A^{F}\hat{Y}+2\hat{V}A^{F}A\hat{Y}+\cdots =\hat{V}LA^{F}BB\hat{Y}$$ (8)

As a result, according to Eqs (6) and (7), the domestic circulation degree can be expressed as:

$$PLVD=\frac{Xd_{GVC}}{VY_{GVC}}=\frac{\hat{V}LLA^{F}B\hat{Y}}{\hat{V}LA^{F}B\hat{Y}}$$ (9)

According to Eqs (6) and (8), the international circulation degree can be expressed as:

$$PLVI=\frac{Xi_{GVC}}{VY_{GVC}}=\frac{\hat{V}LA^{F}BB\hat{Y}}{\hat{V}LA^{F}B\hat{Y}}$$ (10)

#### Gray correlation model

In this paper, the research period is limited to 2008–2014, and the sample size is small, which is exactly in line with the characteristics of gray correlation analysis method based on “small sample and poor information” to explore valuable information. Therefore, this paper adopts the gray correlation model to study the integration degree of the new development pattern of dual circulation and the green development of industry, and the specific application process is as follows.

In the first step, under the division of three major industries, the domestic and foreign circulation degrees are used as the characteristic series, and the data related to the green development of industries are used as the factor series. I denote the characteristic series as *Y*_*0*_(*T*), and denote the factor series as *Xi*(*t*) (*t* = 1, 2,. . ., 7). Where *i* denotes the green development evaluation index of a certain industry of the three industries. The original data are dimensionless, and the initialized series operator (*D*) is used according to the selected data in this paper, and its formula is

$$x_{i}(k)=\frac{x_{i}^{\prime }(k)}{x_{i}^{\prime }(1)}(i=0,1,\ldots ,m;k=1,2,\ldots ,n)$$ (11)

In the second step, the difference between the characteristic series and each factor series is found, and then I find the maximum and minimum values from Δ*(t)*.


$$\Delta_{i}(t)=|Y_{0}(t)-X_{i}(t)|$$

$$minmi\;n|Y_{0}(t)-X_{i}(t)|,maxma\;x|Y_{0}(t)-X_{i}(t)|$$ (12)

In the third step, the number of correlation coefficients is found. Where *ρ* is the resolution coefficient, which is often taken as 0.5.

The fourth step is to find the gray correlation.


$$R_{i}=\frac{1}{7}\sum_{t=1}^{n}R_{i}(t),(t=1,2,\ldots ,7)$$ (13)

*R*_*i*_ is the gray correlation degree. The higher the value is, the higher the correlation is and the stronger the integration is. The classification of correlation level is shown in Table 2.

**Table 2:** Gray correlation classification criteria.

The Degree of Integration	Correlation Level	Correlation Coefficient
Less integration	Lower correlation	(0.00–0.35]
Medium level of integration	Moderate correlation	(0.35–0.65]
Higher degree of integration	Higher correlation	(0.65–0.85]
High level of integration	Highly correlation	(0.85–1.00]

#### Coupled coordination model

Coupling, originally in physics, denotes a phenomenon in which two or more systems or motions interact with each other and thus influence each other. Coordinate refers to the proper cooperation and harmony between a subject or its internal elements [39].

To measure the coupling and coordination between the two systems more accurately, this paper draws on the study of Tao et al. [40] to construct the following model.


$$C=2\sqrt{\frac{U_{1}\cdot U_{2}}{{\left(U_{1}+U_{2}\right)}^{2}}}$$ (14)

Where *C* denotes the degree of systematic coordination relationship between the two systems. *U*_1_ is the degree of the dual circulation pattern, and *U*_2_ is the degree of green development of industry.

Due to the restrictive factors of the formula itself, the measured results can only reflect the relationship between the two interactions and do not reflect the degree of coupling, so the adjustment model is chosen in this paper [41]. The model can not only effectively solve the control problem of the low-coupling system, but also has strong robustness. Its equation is:

$$T=\alpha U_{1}+\beta U_{2}$$

$$D=\sqrt{C\cdot T}$$ (15)

Where, *T* denotes the comprehensive evaluation index of the two systems and takes the values of [0, 1]. *α* and *β* are the adjustment coefficients of the comprehensive evaluation index. In view of the mutual promotion relationship between the dual circulation pattern and industrial green development, they are considered to have the same importance, and both *α* and *β* are taken as 0.5. *D* represents the coupling degree, and the value is between [0, 1].

The criteria for judging the level of coupling between the dual circulation pattern and industrial green innovation are shown in Table 3 [41].

**Table 3:** Judging criteria for the coupling coordination.

Area of Coupling	Coupling Effect Level	Coupling Degree
Unreconciled area	Severe incoordination	0.000–0.100
	Moderate incoordination	0.101–0.300
	Mild incoordination	0.301–0.400
Adaptation area	Verge of incoordination	0.401–0.500
	Reluctant coordination	0.501–0.600
Coupling area	Moderate coordination	0.601–0.700
	Good coordination	0.701–0.900
	Quality Coordination	0.901–1.000

### Source of data

Since the relevant data of the current world input-output tables are currently integrated only up to 2014, and there are a large number of missing values in the database for China’s trade data before 2008. To ensure the continuity, reliability and validity of the data, this paper only explores the indicators from 2008 to 2014. It was a critical period of global economic development, coinciding with the global financial crisis and the subsequent economic recovery. During this period, many countries and regions underwent economic restructuring and policy reforms to adapt to the new economic environment. Therefore, the range of data for this period was chosen to better reveal the dynamic relationship between the new development pattern of the dual circulation and the green development of the industry. A review of the relevant literature for the period of rapid economic development in China from 2008 to 2014 also reveals the existence of a potential dual circulation architecture in China at that time. In addition, analyzing China’s economic system before the dual circulation was proposed can help further clarify the strategic mission of the new development pattern of the dual circulation, and provide theoretical guidance for optimizing the dual circulation pattern and specifying specific initiatives of the dual circulation.

The data in this paper come from the statistical database of WIOD, the National Bureau of Statistics of China, relevant statistical yearbooks of various years, EPS database and the research results of relevant scholars, etc.

Among them, the following data come from China Statistical Yearbook and China Economic Network Statistical Database: land output rate, labor productivity of secondary industry, comprehensive utilization of industrial solid waste, completed investment in industrial pollution control, sulfur dioxide emission per unit area emissions, solid emissions per unit of regional GDP, and data related to the tertiary industry.

The proportion of water-saving irrigation area to effective irrigation area and energy consumption per unit of industrial added value are from Statistical Communiqué of the People’s Republic of China National Economic and Social Development; the scale of investment completed in soil and water conservation and ecological projects this year is from Statistic Bulletin on China Water Activities; the amount of chemical fertilizer used per unit area and the amount of pesticide used per unit arable area are from China Rural Statistical Yearbook; the amount of water used per unit of industrial value added, the total amount of wastewater per unit of GDP and the investment in green science and technology innovation are from Final Account of Ministry of Ecology and Environment of the People’s Republic of China.

The data related to the world input-output tables are from the WIOD database (source: https://www.rug.nl/ggdc/valuechain/wiod/); the domestic and foreign circulation degree referenced from Liu’s study [34].

## Empirical analysis

### Gray correlation analysis

This paper ranks the gray correlation degree. The results are shown in Tables 4 and 5. We can find that the mean value of the gray correlation between the new development pattern of dual circulation and industrial green development reaches 0.759, which indicates that the two systems are not closed systems that maintain their own independent operation, but they have a natural correlation and have formed a mutual penetration and integration situation.

**Table 4:** Gray correlation analysis of domestic circulation and industrial green development.

Industry	Potential Correlation Factors	Gray Correlation	Correlation Level	Ranking
Primary Industry	Pesticide use per unit of arable land area	0.855	Highly	1
	Fertilizer use per unit area	0.852	Highly	2
	The proportion of water-saving irrigation area to the effective irrigation area	0.762	Higher	3
	Soil and water conservation and ecological projects this year to complete the investment scale	0.664	Higher	4
	Land output rate	0.526	Moderate	5
Secondary Industry	Sulfur dioxide emissions per unit area	0.903	Highly	1
	Labor productivity	0.888	Highly	2
	Energy consumption per unit (10,000 yuan) of industrial added value	0.863	Highly	3
	Industrial pollution control completed investment	0.841	Higher	4
	Total wastewater per unit of GDP	0.84	Higher	5
	Comprehensive utilization of industrial solid waste	0.834	Higher	6
	Water consumption per unit of industrial added value	0.826	Higher	7
	Solid emissions per unit of gross regional product	0.747	Higher	8
	Green technology innovation input	0.562	Moderate	9
Tertiary Industry	The proportion of tertiary industry output value to regional GDP	0.849	Higher	1
	The proportion of employed persons in the tertiary sector	0.790	Higher	2
	Tertiary industry labor productivity	0.593	Moderate	3

**Table 5:** Gray correlation analysis of foreign circulation and industrial green development.

Industry	Potential Correlation Factors	Gray Correlation	Correlation Level	Ranking
Primary Industry	Pesticide use per unit of arable land area	0.826	Higher	1
	Fertilizer use per unit area	0.792	Higher	2
	The proportion of water-saving irrigation area to the effective irrigation area	0.735	Higher	3
	Soil and water conservation and ecological projects this year to complete the investment scale	0.582	Moderate	4
	Land output rate	0.528	Moderate	5
Secondary Industry	Sulfur dioxide emissions per unit area	0.886	Highly	1
	Labor productivity	0.851	Highly	2
	Energy consumption per unit (10,000 yuan) of industrial added value	0.840	Higher	3
	Industrial pollution control completed investment	0.803	Higher	4
	Total wastewater per unit of GDP	0.798	Higher	5
	Comprehensive utilization of industrial solid waste	0.781	Higher	6
	Water consumption per unit of industrial added value	0.768	Higher	7
	Solid emissions per unit of gross regional product	0.694	Higher	8
	Green technology innovation input	0.569	Moderate	9
Tertiary Industry	The proportion of tertiary industry output value to regional GDP	0.822	Higher	1
	The proportion of employed persons in the tertiary sector	0.783	Higher	2
	Tertiary industry labor productivity	0.557	Moderate	3

#### Analysis of the correlation between domestic circulation and industrial green development

Overall, it seems that the mean value of the correlation between domestic circulation and industrial green development reaches 0.776, which indicates a high degree of correlation between the two systems.

In the primary sector, pesticide use per unit arable area and fertilizer use per unit area have the strongest correlation with the domestic circulation, and both reach a high degree of correlation. This indicates that a higher influx of intermediate inputs such as pesticides and fertilizers into the production process leads to a significant increase in land resource utilization, and the resilience of the internal circulation chain is consolidated. Along with the integration of agriculture with life science, mechanical science and biological science, the trend of agricultural modernization and transformation is becoming clearer. The distance required for the industry to move from primary elements and raw material supply to final product manufacturing will further increase, and the scale of the industry and its radiation absorption capacity to other industries will be significantly enhanced.

In the secondary industry, the correlation between sulfur dioxide emissions per unit area, labor productivity and domestic circulation is strong. Certain industries have higher sulfur dioxide emissions, such as coal, iron and steel, chemical industry, etc. If we focus on these industries, sulfur dioxide emissions will continue to increase on the one hand, and on the other hand, it will make the length of domestic production increase. However, the domestic circulation can help clarify the allocation of basic resources and guide the domestic basic manufacturing and raw material supply to a refined model. From the aspect of labor productivity, the domestic circulation focuses on supply-side structural reform, which implies the development of high-tech, high value-added, low-resource-consumption and low-pollution industries. Moreover, labor productivity in these industries is usually higher. As the domestic circulation develops, more and more green technologies and clean energy are being used in the production process, which will improve production efficiency and, in turn, labor productivity.

In the tertiary industry, the indicator of the proportion of the output value of the tertiary industry to the regional GDP has a high correlation with the domestic circulation. With the continuous progress of social civilization, consumers’ awareness of environmental protection will continue to improve, and the proportion of green consumption in the tertiary industry will gradually increase. The development of domestic circulation can promote the greening of production, improve product quality, and meet and enhance consumers’ demand for green products.

#### Analysis of the correlation between foreign circulation and industrial green development

Briefly, the mean value of correlation between foreign circulation and industrial green development reaches 0.742, indicating a high degree of correlation between the two systems. Very similarly, pesticide use per unit of arable land area, labor productivity, and the share of tertiary industry output in regional GDP have high correlations with the foreign circulation.

The degree of foreign circulation refers to the link required to circulate the products abroad from primary form to final form. As the times of circulation grows, the technological lifelines of the industry are vulnerable to being controlled by another countries and become subordinate to their industries.

From the perspective of international circulation, this means that while the modernization of agriculture is further clarified by the integration of agriculture with biological and mechanical sciences, the distance required for the industry to move from primary elements to final goods abroad will further increase. This will likely lead to a deepening of China’s import dependence on foreign agricultural products.

In addition, the increase in labor productivity often means the birth of more high-tech, high value-added, low-resource-consumption and low-pollution industries. Similarly, the times of the circulation of such industries abroad will increase. This will make China’s green technologies more easily controlled by other countries and become subordinate to their industries.

Last but not least, with the continuous development of the tertiary sector and the further promotion of the concept of green consumption, the length of the foreign circulation of the tertiary sector will also be further extended. This will make the domestic demand for green imported products further increase.

### Analysis of the coupling degree between new development pattern of dual circulation and industrial green development

#### Coupling and coordination analysis of domestic circulation and industrial green development

The coupling coordination degree of green development and domestic circulation of Chinese industries is shown in Table 6. The coupling degree of green development level of primary industry and domestic circulation degree is good, with a mean value of 0.726, which is in good coordination grade. Since 2010, their coupling degree has shown a steady increase, which shows that the Chinese government has been paying more attention to agricultural development. As a large agricultural country, China has a long-standing trend of internal circulation in agriculture, except for some agricultural products that depend on imports. In this process, the enhancement of the internal circulation trend effectively stimulates agricultural and rural kinetic energy, and combined with the optimization effect of industrial green transformation on agricultural business entities, the two generate a more positive synergy. In addition, the shaping of the internal circulation pattern has led the government to adopt effective incentive policies to prompt multiple subjects to invest in agricultural development and governance. Therefore, the coupling of agricultural inner circulation and industrial green development is an important template for China’s industrial transformation.

**Table 6:** Coupling and coordination degree between domestic circulation and industrial green development.

Industry	2008	2009	2010	2011	2012	2013	2014	Mean
Primary Industry	0.727	0.601	0.637	0.705	0.784	0.801	0.825	0.726
Secondary Industry	0.350	0.450	0.602	0.698	0.844	0.900	0.993	0.691
Tertiary Industry	0.253	0.770	0.846	0.316	0.708	0.758	0.613	0.609

The coupling degree of the secondary industry ranges from 0.350–0.993 with a mean value of 0.691, which is in a moderate coordination state. Compared to 2008, the secondary industry moved from a mildly disordered state to a high-quality coordinated state in 2014. In fact, China’s manufacturing industry has long faced the impact of the overheated virtual economy. However, with the launch of the State Council’s Industrial Transformation and Upgrading Plan (2011–2015), the manufacturing industry has gradually moved toward the development path of green transformation. Undeniably, China’s manufacturing industry has long been at the disadvantage of the global value division of labor, yet it is the pillar industry of the domestic economic circulation system. The former means that China can not give up the vast foreign market, and therefore it will be caught in the quagmire of low-end technology lock; the latter means that China is at a disadvantage in the competition with similar foreign products due to the lack of core technology.

However, the coupling of internal circulation and industrial green development has effectively cracked the contradiction between manufacturing development and green transformation: the innovation of environmental protection technology effectively promotes the green optimization and upgrading of industrial economic structure and realizes the green development of the manufacturing industry. The green transformation of China’s economic development comes from the cultivation of new green industries. Emerging green industries are born out of science and technology innovation. In this circulation facilitated by science and technology innovation, the green transformation efficiency of the manufacturing industry is effectively enhanced, and it is used to meet the needs of the domestic circulation to achieve quality and efficiency within the industry.

The tertiary sector has a coupling degree between 0.253 and 0.613, with a more pronounced overall span. Its mean value is 0.609, which is in a moderate state of coordination. Due to the special nature of service consciousness and national public utilities, the internal circulation is curbed. This makes its coupling degree the lowest among the three industries and showing coupling instability. The 2008 financial crisis brought a huge blow to China’s economic development. The Chinese government, through the steady rise of domestic demand, the innovative adjustment of business models and service ideas, and the introduction of smart industries, has made the coupling degree of the internal circulation of the tertiary industry and the green development of the industry rise again.

#### Coupling and coordination analysis of foreign circulation and industrial green development

From Table 7, we can see that foreign circulation and industrial green development are in a moderate coupling state. The primary industry and the secondary industry both enter the coupling zone, with the average value of 0.611 and 0.746 respectively. The tertiary industry is in the adaptation zone, with an average value of 0.528, and is in the primary coordination state.

**Table 7:** Coupling and coordination degree between foreign circulation and industrial green development.

Industry	2008	2009	2010	2011	2012	2013	2014	Mean
Primary Industry	0.792	0.627	0.503	0.440	0.456	0.581	0.882	0.611
Secondary Industry	0.430	0.599	0.769	0.769	0.824	0.891	0.939	0.746
Tertiary Industry	0.183	0.313	0.838	0.813	0.184	0.884	0.481	0.528

China is already concerned about the potential crisis in the international market and clearly understands that green development is an inevitable requirement to improve international competitiveness. Currently, countries around the world are focusing on new energy and energy conservation and environmental protection as the focus of the next round of industrial development. As a developing country, China must also develop a green economy to form a new economic growth point and win a place in the international market.

During the window period of the post-crisis era, China circumvented its over-dependence on foreign technology by fostering the acceleration of industrial environmental technology innovation, unblocking the interface between technology, finance and real industry, and opening up the foreign circulation of production, distribution, exchange and consumption.

### Coupling type analysis

Based on the difference between the dual circulation degree and the industry green development degree (Δ*U* = 0, Δ*U* = *U*_1_ ‒ *U*_2_) and the need for standardization, this paper refers to Wei’s division criteria [42], the coupling degrees of both are further divided into three categories. When Δ*U*>0.225, the coordination type is circulation leading state (S, i.e., green development lag state); when Δ*U* is in the interval of [0.155, 0.255], it behaves as a synergistic co-progressive state (T, i.e., green development stable state); when Δ*U*<0.155, it enters a circulation lag state (F, i.e., green development leading state).

#### Domestic circulation and industrial green development coupling type

The coupling results are shown in Table 8. Among the coupling types of domestic circulation and industrial green development of each industry in China, F-type is predominant, which indicates that industrial green development has a positive driving influence on promoting domestic circulation, reflecting to some extent the driving nature of industrial green development.

**Table 8:** Types of coupling between domestic circulation and industrial green development in major years.

Industry	2008			2010			2012			2014		
	ΔU	D	Type	ΔU	D	Type	ΔU	D	Type	ΔU	D	Type
Primary Industry	0.321	0.727	S	0.161	0.637	T	0.309	0.784	S	0.061	0.825	F
Secondary Industry	0.262	0.350	S	-0.017	0.602	F	0.105	0.844	F	0.028	0.993	F
Tertiary Industry	0.031	0.253	F	0.754	0.846	S	-0.322	0.708	F	-0.784	0.613	F

The primary industry, along with the green transformation of agriculture in full swing, the leading role of industrial green development gradually came to the fore, but due to the problem of technical barriers in the green transformation, the drive of industrial green development dropped back in 2012 and showed an upward trend in 2014.

In the secondary industry, as the industrial greenization transition was in its infancy in 2008, and at the same time affected by the financial crisis, it presents a green development lag (S). From 2010 onwards, it gradually shows a green development drive dynamic (F). Thus, it can be seen that since the financial crisis, China began to pay more attention to relying on greenization and supply-side structural reform to lead the change of the domestic circulation system. At that time, Li argued that the basic contradiction of China in the future was the continuous growth of production capacity under the high savings rate and the relative lack of domestic demand. An effective solution to this contradiction was to carry out a green industrial revolution and upgrade the existing production capacity [43]. The report of the 18th National Congress of the Communist Party of China in 2012 clearly proposed that “vigorously promote the construction of ecological civilization”, make efforts to reverse the deterioration of ecological environment, and integrate the construction of ecological civilization into all aspects and the whole process of political, economic, cultural as well as social construction. Although faced with the deteriorating economic situation at home and abroad and the enormous pressure of industrial transformation and upgrading, China has vigorously promoted the supply-side structural reform and resolutely won the “Blue Sky Defense” in order to achieve sustainable development.

The tertiary sector shows a non-stable state with alternating F-state and S-state. The reason for this may lie in the structural imbalance of China’s service industry development. Since the post-economic crisis era, industries in various countries have stagnated and the contradiction between supply and demand has become increasingly evident. Although China, as the world’s second largest consumer, has shown a certain degree of economic resilience, its internal factor allocation distortions have long existed. At that time, the attractiveness of consumer goods in western developed countries to Chinese consumers remained strong, while the upgrading of domestic consumer goods in China was restricted, which led to the S-state of China’s tertiary industry in 2010. With the optimization of the supply structure and the market dynamics brought by innovation development, China’s domestic market has become significantly more adaptable and flexible to changes in demand, thus pulling the service sector into the F-state.

#### Foreign circulation and industrial green development coupling type

The coupling results are shown in Table 9. China’s foreign circulation and green development degrees gradually show green development leading state (F) in the secondary and tertiary industries. However, the primary industry shows alternating driven state.

**Table 9:** Types of coupling between foreign circulation and industrial green development in major years.

**Industry**	**2008**			**2010**			**2012**			**2014**		
	**ΔU**	**D**	**Type**	**ΔU**	**D**	**Type**	**ΔU**	**D**	**Type**	**ΔU**	**D**	**Type**
Primary Industry	0.608	0.792	S	-0.140	0.503	F	-0.390	0.456	F	0.279	0.882	S
Secondary Industry	0.662	0.430	S	0.572	0.770	S	0.034	0.824	F	-0.172	0.939	F
Tertiary Industry	0.999	0.183	S	0.592	0.838	S	-0.524	0.184	F	-0.878	0.481	F

The primary industry showed a shift from S-state to F-state and back to S-state, which indicates that China’s demand for foreign agricultural products was larger in 2008 due to the financial crisis. In order to adapt to the new international and domestic situation and new development China responds to the development path of “green agriculture” [44]. At that time, China continued to develop science and technology and introduced advanced industrial equipment and advanced management concepts. The standardization of agricultural products was advocated as a means to promote comprehensive, coordinated and sustainable social and economic development. However, in 2014, the coupling type shifted back to S-type, which may be due to natural disasters or other endogenous factors, but it was also short-lived.

In the secondary industry, the coupling type reflects the trend that foreign circulation and industrial green development are gradually moving closer to the green development leading state (F). This is mainly attributed to the adjustment of China’s industrial structure, the rapid development of green and low-carbon industries and the year-on-year increase in industrial exports.

For the tertiary industry, the trend is similar to that of the secondary industry, which is also in a state of transition from S to F. With the change of new industries, the innovation of core models and strategic thinking of China’s service industry has been strengthened, and the integration of innovative technology and service paths has become the focus of industrial adjustment under the current new development pattern of dual circulation.

## Conclusions and recommendations

### Conclusions

Through the gray correlation analysis, we found that the average correlation between domestic and international circulation and industrial green development reached above 0.7, i.e., a high correlation level. Specifically, the two systems have a high correlation in four aspects—pesticide use per unit arable land area, sulfur dioxide emission per unit area, labor productivity, and the proportion of tertiary industry output value to regional GDP.

From the point of view of coupling degree, the domestic circulation and industrial green development show positive and benign interaction, and the overall coupling degree is high, basically crossing the adaptation zone into the coupling state. However, as far as the internal industry is concerned, there is a collapse problem in the tertiary industry. The changing trend of international circulation is basically the same as that of domestic circulation. Among them, the primary industry and the secondary industry both enter the coupling zone; the tertiary industry has the problem of fluctuating coupling, and the mean value of coupling degree is low, in the state of reluctant coordination.

In terms of coupling types, on the one hand, the domestic circulation degree and the level of industrial green development basically show a green development leading state (F), that is, industrial green development drives domestic circulation, especially in the secondary industry. The coupling of the tertiary industry is more volatile and strongly influenced by external factors, which may trigger the shrinkage of the domestic market. On the other hand, the international circulation and green development degrees gradually show a green development leading state (F) in the secondary and tertiary industries in major years. However, due to the financial crisis or natural disasters, the primary industry shows alternating leading state.

### Recommendations

#### Coordinate the development of the industry internally and externally

Coordination within the industry means that the green development of the industry should integrate the three levels of efficiency, scale and equity. Even though the efficiency of industrial development is the goal actively pursued by any industrial sector and the main source of national economic increment, the unilateral pursuit of efficiency and neglect of scale and equity indicators will have a negative impact on industrial green development. By the same token, a unilateral focus on scale and equity will not achieve the purpose of economic development. In the eyes of developed countries, industrial green development requires "de-industrialization", but China’s industrial green development should be a more developed technical concept, that is, to coordinate the three levels of development needs of economic efficiency, moderate scale and social equity, to coordinate the three major layouts of production, living and ecology, and to improve the sustainability of the region’s economy and livability of the city.

External coordination of industry refers to the division of labor and cooperation and orderly competition among regional industries to achieve coordinated development of regional industries. The green development of industries in neighboring regions has significant spatial dependence, which to a certain extent indicates the convergence of leading industries in neighboring regions. The coordination of economic development between regions is the key to realizing green development. On the one hand, inter-regional industries should strengthen the sense of cooperation, and regions located in low agglomeration areas need to comply with the structural reform on the supply side, fully consider the allocation utility of market resources, actively digest production capacity, bring into play total factor productivity, and achieve intensive growth. The regions located in high aggregation areas should seize the opportunity of "One Belt, One Road" construction, fully utilize their own advantages in location, industry and human capital to realize the flow and diffusion of technology, capital, information and other factors. On the other hand, inter-regional industries can engage in healthy competition, focusing on intellectual property protection while also encouraging active cooperation and sharing of practical experience.

#### Take innovation as the driving force to promote the green transformation of industries

In the 21st century, with the rapid development of new-generation information technology such as the Internet of Things, cloud computing, big data, mobile Internet and artificial intelligence, Germany proposed the concept of "Industry 4.0" in 2011. In the context of Industry 4.0, innovation has become the driving force for sustainable human development [45], and industrial innovation has become the first driving force of industrial upgrading.

As mentioned above, China has long been trapped in the "low-end lock-in" effect, which on the one hand has a great negative impact on resources and the environment, and on the other hand, has caused overcapacity and inefficient resource allocation. In order to achieve green development, China’s industry should take advantage of this economic and social change to achieve green transformation with innovation as the driving force and technology as the guide.

First, promote the deep integration of industry and the Internet economy, integrate information resources and other elements, and consolidate and strengthen the core power of industrial green development. Second, increase the proportion of service and high-tech industries, optimize the industrial structure, and keep the utilization rate of ecological resources in a reasonable range. Third, drive the wave of science and technology with innovation, optimize the supply resources and improve the level of industrial division of labor.

In addition, the EU in its published report mentioned six enabling technologies for Industry 5.0, namely personalized human-computer interaction, bionic technology and smart materials, digital twin and simulation, data transmission, storage and analysis technologies, artificial intelligence, and energy efficiency, renewable energy, storage and autonomy technologies [46]. This should also be the direction of China’s future science and technology innovation based on sustainable development orientation.

#### Take green sharing as the goal to strengthen the policy orientation of green development

With the rapid development of the new generation of information technology and its deep integration with industry, based on the current background of global warming, increasing environmental pollution, continuous consumption of resources and energy and increasing pressure of industrial competition, the concept of Industry 5.0 has been proposed in Europe to describe the vision of industrial development and as a solution needed for human beings to pursue social prosperity and stability. The first EU white paper on Industry 5.0 mentions that Industry 5.0 has three characteristics: human-centeredness, sustainability, and resilience [47].

Green has become a necessary condition for sustainable development, and it is also necessary to form the harmonious development of humans and nature and promote the construction of beautiful China, while sharing is the essential requirement of socialism with Chinese characteristics. The government is the core force to guide and encourage the green development of industries, and it is also the "visible hand" for the implementation of macro strategies. The government needs to introduce relevant policies and measures to encourage and guide the green development of industries.

First, adhere to people-oriented, pay attention to social issues and return to people-centered value orientation; establish an environmental governance system and ecological compensation system to create a good environment for industrial green development. Second, adhere to sustainable development-oriented, the introduction of relevant green industrial policy, on the one hand, through environmental regulation to stimulate industrial innovation, with technological revolution to change industrial non-clean production; on the other hand, improve the ecological civilization performance assessment and evaluation and accountability system, and clarify the social responsibility of enterprises to discharge and treat pollution. Thirdly, ride on the tailwinds of new industrialization and informationization, establish a spatial planning system, reasonably plan regional development, and formulate development strategies for modern service industries and high-tech industries, so that industries can achieve structural green development.

#### Take the opportunity of mutual promotion of dual circulation to stabilize the steady state of green development coupling

Based on the information capture and industrial linkage of the international circulation, timely adjustment of the development direction of the domestic circulation, thus truly empowering the high-quality development of the domestic economy, is the key opportunity for industrial transformation brought by the dual circulation pattern. Therefore, each industry should strive to integrate itself, make the domestic market become a gravitational field and a platform for international factors to gather, and win the strategic high ground of the world’s future development with advanced deployment.

Dialectical and complete view of the domestic and foreign dual circulation pattern, and industrial green development into the dual circulation system, in order to achieve holistic guidance. Firstly, the domestic circulation must be guided to deeply integrate into the international circulation to ensure the autonomy, ecology and integrity of the domestic circulation in order to counteract potential international competition in the future; secondly, the international circulation should be guided to provide competitive incentive thinking for the domestic circulation, so as to lay the development momentum and frontier elements for the high quality and high-level development of the domestic circulation.

On the basis of the formation of the dual circulation pattern, drive the industry green development system effectively embedded in it, firstly, to ensure the promotion of green development by the dual circulation, and then, as far as possible, play the coupling leading power of green development, but not overly pursue green improvement and ignore the efficiency of the cycle chain. Secondly, the green leading steady state should not be pursued excessively in the international circulation, and the green operation experience of the world industrial chain, value chain and supply chain should be learned through the coordinated co-progressive model as far as possible and introduced into the domestic circulation system.

## Supporting information

S1 Data (XLSX)

S2 Data (XLSX)

S3 Data (XLSX)

S4 Data (XLSX)
